# Calcineurin inhibitors reduce NFAT‐dependent expression of antifungal pentraxin‐3 by human monocytes

**DOI:** 10.1002/JLB.4VMA0318-138R

**Published:** 2019-04-01

**Authors:** Kamila Bendíčková, Federico Tidu, Marco De Zuani, Marcela Hortová Kohoutková, Ivana Andrejčinová, Antonio Pompeiano, Silvie Bělášková, Giancarlo Forte, Teresa Zelante, Jan Frič

**Affiliations:** ^1^ International Clinical Research Center St. Anne's University Hospital Brno Brno Czech Republic; ^2^ Department of Biology Faculty of Medicine Masaryk University Brno Czech Republic; ^3^ Department of Experimental Medicine University of Perugia Perugia Italy

**Keywords:** antifungal response, cyclosporine A, pattern recognition receptor signaling, Tacrolimus

## Abstract

Calcineurin (CN) inhibitors are effective clinical immunosuppressants but leave patients vulnerable to potentially fatal fungal infections. This study tested the hypothesis that CN inhibition interferes with antifungal immune defenses mediated by monocytes. We showed that NFAT is expressed by human monocytes, and is activated by exposure to fungal ligands. We confirmed that NFAT translocation potently activated target gene transcription using a human monocytic reporter cell line. Inhibition of CN‐NFAT by cyclosporine A significantly reduced monocyte production of TNF‐α, IL‐10, and MCP‐1 proteins in response to pattern recognition receptor ligands as well as to *Aspergillus fumigatus* conidia. Moreover, we revealed that human monocytes express the antifungal protein pentraxin‐3 under control of NFAT. In conclusion, clinical CN inhibitors have the potential to interfere with the novel NFAT‐dependent pentraxin‐3 pathway as well as antifungal cytokine production in human monocytes, thereby impeding monocyte‐mediated defenses against fungal infection in immune‐suppressed patients.

## Graphical abstract

NFAT is expressed by human monocytes and regulates expression of the antifungal protein pentraxin‐3 in response to stimulation by pathogen‐derived molecules.

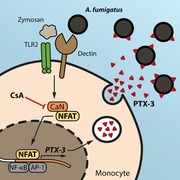

**Abbreviations**
CN: calcineurin
CNS: Conserved noncoding sequence
CsA: cyclosporine A
DCs: dendritic cells
GvHD: Graft‐versus‐host disease
HSCT: hematopoietic stem cell transplantation
PRRs: pattern recognition receptors
PTX‐3: pentraxin‐3
ROI: region of interest
UTR: untranslated region

## 1 INTRODUCTION

Immuno‐suppressive drugs, such as cyclosporine A (CsA) and tacrolimus, are effective in preventing the rejection of solid organ transplants,^1^ reducing graft‐versus‐host disease (GvHD) after hematopoietic stem cell transplantation (HSCT),^2^, ^3^ and treating autoimmune disorders.^4^ However, by down‐regulating the immune response these agents also leave patients susceptible to infections,^5^ particularly by fungal pathogens. Severe/invasive fungal infections occur in ∼3% of post‐transplant patients per year, leading to death in almost 40% of affected patients.^6^, ^7^, ^8^ Thus there is an urgent need to understand the interactions of immune‐suppressive drugs with key components of the host's immune system, both in terms of how they execute their therapeutic effect and the mechanisms underpinning the increased susceptibility of patients to potentially fatal infections.

Tacrolimus and CsA are calcineurin (CN) inhibitors that work by preventing the CN‐dependent de‐phosphorylation of four functionally overlapping members of the NFAT family of transcription factors, thereby preventing NFAT nuclear translocation and transcription of proinflammatory target genes. The NFAT family was originally characterized as a key regulator of T‐cell functions; however, recent data have revealed important roles of the NFAT family in dendritic cells (DCs) and macrophages, which together cooperate as APCs to initiate, shape, and maintain protective immune responses to pathogens.^9^ Initial studies in murine DCs identified NFAT as the key regulator of DC IL‐2 expression,^10^, ^11^ which is crucial to modulate Th17 responses: mice lacking IL‐2 in DCs express higher levels of IL‐17 in their lungs, leading to a pathologic Th17 response that results in exacerbated infection by the fungal pathogen *Aspergillus fumigatus* (*A. fumigatus*).^12^ A similarly important role for IL‐2 expressed in myeloid cells was shown as crucial to maintain gut homeostasis.^13^ Alongside NFAT regulation of IL‐2, genome‐wide mapping of NFAT1 binding sites in murine DCs activated by the fungal beta‐glucan, curdlan, identified a panel of inflammatory cytokines, including TNF, IL‐10, and GM‐CSF, that is regulated under the control of the NFAT1 binding promoter.^14^ Similarly, murine macrophages impaired in CN exhibit reduced responses to LPS both in vitro and in vivo, producing lower levels of IL‐12 and IL‐23,^15^, ^16^ and defects in fungal killing that were associated with increased mortality from pulmonary aspergillosis.^17^ Recent evidence also confirms a clear role for CN–NFAT in human macrophages in intracellular restriction of *A. fumigatus* infection in vitro.^18^, ^19^

NFAT activation in DCs and macrophages can occur following ligation of several pattern recognition receptors (PRRs), including the TLR4–CD14 complex, which detects bacterial LPS, with the crucial role of CD14 in inducing the NFAT activation,^20^ TLR9,^21^ which binds microbial DNA, and dectin‐1,^14^, ^22^ which recognizes fungal cell wall glucans. Of these receptors, dectin‐1 is the most crucial during fungal infections: human HSCT patients carrying a dectin‐1 polymorphism with lower receptor activity face a higher risk of aspergillosis, which correlates with reduced expression of IFN‐γ, IL‐10, and IL‐17A by their peripheral blood monocytes.^23^ Dectin‐1 ligation is known to trigger expression of number of antifungal cytokines including IL‐1β, IL‐6, IL‐23^24^, whereas CN inhibition in murine models has further identified IL‐2,^12^ IL‐10,^25^ TNF‐α^21^, and more recently pentraxin‐3 (PTX‐3)^26^ as having notable effects on *A. fumigatus* and *Candida albicans* susceptibility. Of particular interest is the emerging role of PTX‐3, a soluble PRR with critical functions in antifungal immunity: impaired PTX‐3 production directly increases susceptibility to *A. fumigatus* infection in mouse models^27^ and human HSCT patients.^28^ Within myeloid cells, PTX‐3 is produced by both mononuclear phagocytes responding to inflammatory or infectious stimuli^29^ and neutrophils, which produce PTX‐3 during granulocytic differentiation and store it in a form of ready‐to use cytoplasmic granules.^30^ Once released from cells, PTX‐3 recognizes and binds fungal conidia, which then activate neutrophil phagocytic activity and complement to clear the infection.^31^ PTX‐3 expression has previously been associated with several regulatory molecules and transcription factors including Pu1, SP1, NF‐κB, AP‐1, and NF‐IL‐6.^32^ We also recently reported that CN‐deficient murine DCs and LysM^+^ myeloid cells exhibit significantly reduced Ptx‐3 expression,^26^ suggesting a possible link between CN–NFAT signaling and Ptx‐3 expression in murine myeloid cells.

Many studies have now been conducted on the roles of NFAT in murine DCs in particular, but there is a dearth of knowledge on the potential roles of the CN–NFAT pathway in other myeloid cell types, and in human cells in particular. Given the emerging data on the significance of the monocytic compartment in determining human susceptibility to aspergillosis,^23^ we aimed to establish whether the NFAT pathway is active in human monocytes; the effects of CN inhibition on these cells; and whether there was a direct link between PRR ligands, NFAT, and production of antimicrobial molecules by human monocytes. We reveal that human monocytes do express functional NFAT family members and that inhibition of CN–NFAT signaling profoundly affects the expression of key inflammatory cytokines in these cells. Moreover, PRR ligation as well as stimulation with *A. fumigatus* conidia induce PTX‐3 expression in human monocytes, which is significantly decreased by CN–NFAT inhibition. In the context of these findings the fact that CsA treated monocytes are significantly impaired in PTX‐3 expression is of high clinical relevance considering that *A. fumigatus* infections are serious life‐threatening complications in patients undergoing bone marrow transplantations.

## 2 MATERIALS AND METHODS

### 2.1 Isolation and stimulation of human blood monocytes

Monocytes from healthy donors were isolated from fresh buffy‐coats (Department of Transfusion & Tissue Medicine of the Brno University Hospital, Brno, Czech Republic) using RosetteSep Human Monocyte Enrichment Cocktail (STEMCELL Technologies, Vancouver, Canada). Untouched monocytes were isolated by gradient centrifugation using Lymphoprep (density 1.077 g/ml; STEMCELL Technologies) following the manufacturer's recommendations. Monocytes were resuspended in X‐VIVO 15 medium (Lonza, Basel, Switzerland) without supplementation and seeded at a concentration of 2 × 10^6^ cells/ml into cell culture plates (Thermo Fisher Scientific, Waltham, MA, USA). Monocytes were pre‐treated with 1 μg/ml CsA (Cell Signaling Technology, Danvers, MA, USA) for 1 h before exposure to either 1–5 μg/ml zymosan (InvivoGen, San Diego, CA, USA), 1 μg/ml LPS‐EB (LPS from *E. coli* O111:B4) (InvivoGen), 100 ng/mL Pam3CSK4 (InvivoGen), 1 μg/mL curdlan (Megazyme, Chicago, IL, USA), or to heat inactivated *A. fumigatus* (MYA‐4609; ATCC, Manassas, VA, USA) conidia (MOI 4) for 4 h and/or 18 h. *A. fumigatus* conidia were harvested in PBS/0.05% Tween 20 after 5 d of cultivation on potato dextrose agar (Sigma Aldrich, St. Louis, MO, USA).

### 2.2 Immunofluorescence labelling of monocytes

Monocytes or their sorted subsets were seeded into μ‐Slide VI 0.4 (IBIDI) at a concentration of 0.7–1 × 10^6^/ml and incubated for 1 h (37°C, 5% CO_2_). Cells were washed in PBS and fixed with 4% formaldehyde for 15 min at 4°C. BSA (2.5%, Santa Cruz Biotechnology, Dallas, TX, USA) was used for blocking. Samples were incubated overnight with anti‐PTX‐3 (ab90806, Abcam, Cambridge, UK), anti‐CD14 biotinylated (eBiosciences) and anti‐NFAT1 (Cell Signaling Technologies) antibodies. For detection, secondary antibodies AF488 Donkey anti‐rabbit and AF555 Goat anti‐rat (Thermo Fisher Scientific) and streptavidin AF‐647 (eBiosciences, Thermo Fisher Scientific) were used. All antibodies were diluted in DAKO Antibody diluent (DAKO). DAPI (Sigma Aldrich) was used as a nuclear counterstain. Samples were mounted in Mowiol 40–88 (Sigma Aldrich) and images were captured under a Zeiss LSM 780 confocal microscope fitted with a 40 (1.3 numeric aperture) oil‐immersion objective. Image processing was performed in FIJI.^33^

### 2.3 Flow cytometry‐based cell sorting

To obtain highly enriched populations of monocytes, we first excluded cells expressing the lineage markers CD3, CD19, CD20, CD56, CD66b, and CD235α. Cells were labelled with biotinylated primary antibodies (BioLegend, San Diego, CA, USA), followed by streptavidin BV510. Monocyte subsets were then sorted from the Lin^−^ HLA‐DR^+^ population as follows: classical (CD14^+^CD16^−^), intermediate (CD14^+^CD16^+^), and nonclassical (CD14^lo^CD16^+^),^34^ using CD14‐PE, CD16‐APC, and HLA‐DR‐FITC antibodies (eBiosciences). We confirmed the purity of the monocyte subsets (average 97%, 77%, and 90% for classical, intermediate, and nonclassical, respectively) and viability (typical viability > 90%) using MOFLO Astrios (Beckman Coulter, Brea, CA, USA).

### 2.4 Evaluation of PTX‐3 expression in monocytes

Monocytes labelled for surface marker expression were fixed and intracellularly labelled for PTX‐3 (ab125007, Abcam) using an Intracellular Fixation and Permeabilization Buffer Set (eBiosciences) and secondary antibody AF488 donkey‐anti‐rabbit (Thermo Fisher Scientific). Sample acquisition was performed using a FACS Canto II (BD Biosciences, Franklin Lakes, NJ, USA) and the data were analyzed using FlowJo v.10.

### 2.5 RNA extraction and quantitative real‐time PCR

Total cellular RNA was extracted from monocytes or monocyte subsets using Trizol LS reagent (Thermo Fisher Scientific) following manufacturer's recommendations. Samples were then centrifuged at 12,000 ×*g* for 15 min at 4°C. The upper aqueous phase was collected and mixed with an equal volume of 70% ethanol and mixture was transferred to RNeasy spin column (Qiagen, Hilden, Germany). Subsequent RNA purification was performed as recommended by manufacturer. The RNA concentration and quality were determined spectrophotometrically using Nanodrop (Agilent, Santa Clara, CA, USA). RNA was immediately transcribed into cDNA using the high‐capacity cDNA Reverse Transcription Kit (Thermo Fisher Scientific). Real‐time PCR (qPCR) was carried out with Taqman probes (Taqman Gene Expression Assay, Thermo Fisher Scientific) using TaqMan Gene Expression Master Mix (Thermo Fisher Scientific). qPCR analysis was performed using a LightCycler II (Roche, Basel, Switzerland). The Ct values of genes of interest were normalized to house‐keeping gene GAPDH (ΔCt) and the relative expression of each gene of interest was calculated as 2^−ΔCt^. The following Taqman probes were used: NFAT1 (Hs00905451_m1), NFAT2 (Hs00542678_m1), NFAT3 (Hs00190037_m1), NFAT4 (Hs00190046_m1), NFAT5 (Hs00232437_m1), PTX‐3 (Hs00173615_m1), IL‐6 (Hs00174131_m1), and GAPDH (Hs02758991_g1).

### 2.6 ELISA

The commercial DuoSet ELISA (R&D Systems, Minneapolis, MN, USA) was used for detection of PTX‐3, TNF‐α, MCP‐1, and IL‐6 in supernatants. ELISA were performed exactly as recommended by the manufacturer.

### 2.7 Multiplex bead‐based assay for cytokine and chemokine quantification

Simultaneous quantification of 13 human inflammatory cytokines/chemokines, including IL‐1β, IFN‐α2, IFN‐γ, TNF‐α, MCP‐1 (CCL2), IL‐6, IL‐8 (CXCL8), IL‐10, IL‐12p70, IL‐17A, IL‐18, IL‐23, and IL‐33 in supernatants was carried out using the LEGENDplex Human Inflammation Panel (BioLegend), as recommended by the manufacturer. Sample acquisition was performed using a FACS Canto II (BD Biosciences) and the data were analyzed using LEGENDplex Data Analysis software V8.0 (VigeneTech).

### 2.8 Establishing the luciferase reporter THP‐1 line and luciferase assay

Human monocytic THP‐1 cells were seeded in RPMI 1640 (Lonza) without antibiotics (5 × 10^5^/mL). The cells were transfected using Cignal Lenti NFAT Reporter (luc) (Qiagen) and cultivated overnight (37°C, 5% CO_2_). Afterward, the medium was removed and the cells were cultivated in complete low glucose RPMI 1640 (Lonza, glucose 1 g/L) for 2 d. Subsequently, puromycin selection (0.5 μg/ml, Santa Cruz Biotechnology) was performed to obtain a homogeneous population carrying the reporter.

The THP‐1 NFAT reporter line was maintained in RPMI 1640 (Lonza) before subsequent stimulation. The cells were pre‐treated with CsA to inhibit NFAT translocation (1 μg/ml, Cell Signaling Technologies) for at least 30 min before stimulation with ionomycin (1 μg/ml, Sigma Aldrich). Luciferase activity was detected 6 h after triggering using the ONE‐Glo Luciferase Assay System (Promega, Madison, WI, USA).

### 2.9 NFAT translocation in THP‐1 and monocytes and cytospin preparation

THP‐1 cells were maintained in RPMI 1640 with 10% FBS (Sigma Aldrich) and peripheral blood monocytes were cultured in X‐VIVO 15 (Lonza) without any supplementation at 37°C in 5% CO_2_ atmosphere. The cells were seeded at 1 × 10^6^/ml into the plate (THP‐1) or μ‐Slide VI 0.4 (IBIDI; primary monocytes) and stimulated with ionomycin (1 μg/ml, Sigma Aldrich) for 30–90 min in the presence or absence of CsA (1 μg/ml, Cell Signaling Technologies). THP‐1 cells were then harvested and spun to generate a cytospin preparation, which were fixed by incubation in 4% formaldehyde for 15 min at 4°C. Primary monocytes were fixed in 4% formaldehyde immediately in a μ‐Slide VI 0.4 chamber. BSA (2.5%, Santa Cruz Biotechnology) was used for blocking. Samples were incubated overnight at 4°C with anti‐NFAT1 (#4389, Cell Signaling Technologies) and biotinylated anti‐CD14 (eBiosciences) antibodies that were subsequently detected using AF488 donkey anti‐goat (Thermo Fisher Scientific) and AF647 streptavidin (eBiosciences) secondary antibodies. All antibodies were diluted in DAKO Antibody diluent (DAKO). DAPI (Sigma Aldrich) was used as a nuclear counterstain. Samples were mounted in Mowiol 40–88 (Sigma Aldrich). Images were acquired using a Zeiss LSM 780 confocal microscope with ×40 (1.3 numeric aperture) oil‐immersion objective. Image processing was done using FIJI.^33^

### 2.10 Calcium flux analysis

Cells were stained at 37° in RPMI 1640 containing Calcium Sensor Dye eFluor 514, 5 μM (Thermo Fisher Scientific). After 30 min, media with dye was removed and the cells were incubated for 30 min in RPMI 1640 at 37°C, 5% CO_2_. Calcium imaging was accomplished using a Zeiss LSM 780 confocal microscope, with a 488 laser in live mode at 37°C, 5% CO_2_. Injection of triggers or media alone (negative control) was performed during live imaging. Each movie recorded 1 frame/second for a total of 4 min (approximately 20 s before and 220 s after the injection of the trigger). Analysis of the fluorescence intensity of each frame was done using FIJI.^33^

### 2.11 Statistics

Graphpad Prism software v.6 was used for statistical analysis. Data were tested for normal distribution and parametric or nonparametric statistical tests were applied as appropriate. Statistical tests used are specified in the figure legends. *P* values <0.05 were considered statistically significant.

### 2.12 Bioinformatics analysis

Gene expression microarray and ChIP‐seq data were retrieved from NCBI's GEO through series accession numbers GSE59896 and GSE59998. Data were loaded with GEOquery^35^ and limma R^36^ packages from the Bioconductor project, in R 3.4.3 language and environment.

Whole genome comparative analyses were performed with VISTA browser^37^ using the Mouse NCBI/mm9 (Jul. 2007) genome as reference. Sequence homology scores were calculated on a 100 bp window. Regions of interest (ROIs) were selected on the basis of the results from ChIP‐seq data reanalysis and the murine ROI3 was assessed for the presence of NFAT1 binging sites with LASAGNA‐search 2.0^38^ using JASPAR CORE matrices. NFAT1 binding matrix was obtained from JASPAR 2018^39^ and *P* values for NFAT1 binding on the murine and human sequences were calculated by LASAGNA‐search 2.0.

## 3 RESULTS

### 3.1 NFAT expression and translocation in human myeloid cells

**Figure 1:**
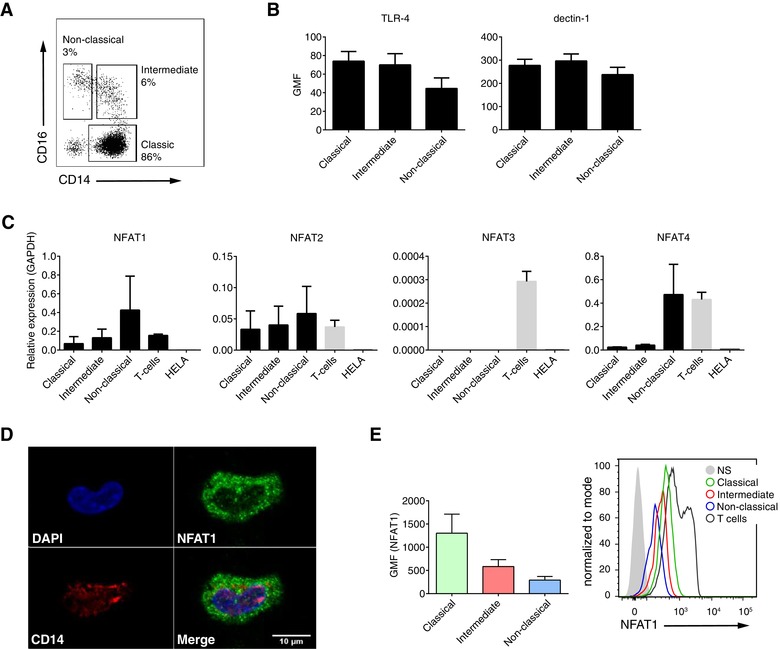
**NFAT expression in human monocytes**. **A**) Monocyte subsets defined based on expression of CD14 and CD16 in the Lin^−^ (CD3, CD19, CD20, CD56, CD66b, CD253α) HLA‐DR^+^ cell population within peripheral blood from 3 healthy donors. **B**) Flow cytometry analysis of intensity of TLR‐4 and dectin‐1 expression on human monocyte subsets. Data are presented as geometric mean of fluorescence (GMF). **C**) Expression of NFAT1‐4 on mRNA level in human monocyte subsets. Peripheral blood T‐cells and HELA cells were used as a positive and negative controls, respectively. **D**) Representative fluorescence microscopy image of NFAT1 expression in CD14^+^ human monocytes. **E**) Flow cytometry measurement of NFAT1 expression on human monocyte subsets

To elucidate the potential contribution of CN–NFAT to human monocyte functions, we first asked whether NFAT‐activating receptors and NFAT family members are expressed in human peripheral blood monocytes from healthy donors. We isolated monocytes as Lin^−^ (CD3, CD19, CD20, CD56, CD66b, CD235α) MHC‐II^+^ cells and separated them by FACS into the 3 conventional monocyte subsets based on their differential expression of CD14 and CD16^34^, ^40^: CD14^+^CD16^−^ classical monocytes, CD14^+^CD16^+^ intermediate monocytes, and CD14^lo^CD16^+^ nonclassical monocytes (Fig. 1A). We then confirmed the expression of the NFAT‐activating PRRs, TLR4, and dectin‐1 using flow cytometry (Fig. 1B), and the gene expression of the NFAT family member genes (Fig. 1C), with T cells and HELA cells as positive and negative controls for NFAT expression, respectively. Monocyte subsets expressed comparable levels of TLR4 and dectin‐1 (Fig. 1B). We also detected transcripts for NFAT1, NFAT2, and NFAT4 in all monocyte subsets (Fig. 1C), but transcripts for NFAT3 were only present in T cells. NFAT1 was the most abundantly expressed, and we confirmed its expression at the protein level by fluorescence microscopy (Fig. 1D) and flow cytometry (Fig. 1E).

**Figure 2:**
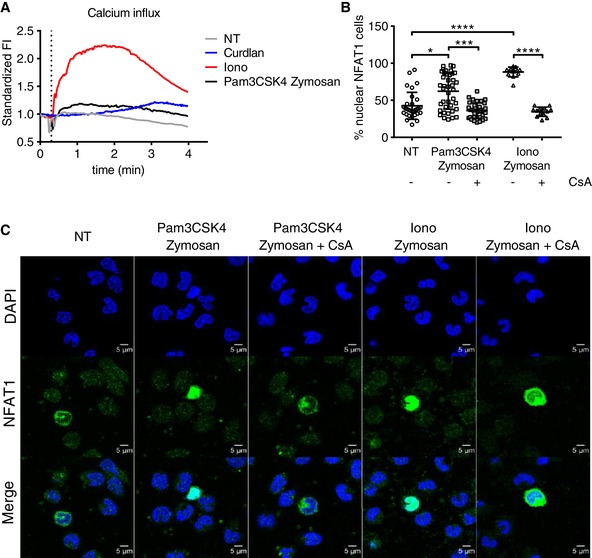
**Functionality of the CN–NFAT pathway in primary human monocytes**. **A**) Calcium flux after ionomycin or curdlan (dectin‐1 ligand) treatment of primary human monocytes. **B** and **C**) Fluorescence microscopy images of primary human monocytes showing NFAT1 translocation from the cytoplasm to the nucleus 45 min after treatment with Pam3CSK4 and zymosan or with ionomycine and zymosan in the presence or absence of cyclosporine A. The inhibitor was administered 60 min prior to the trigger. Quantification of nuclear translocation was performed using ImageJ software and a nuclear/cytoplasmic ratio plug‐in

The activation of target gene transcription by NFAT occurs downstream of PRR ligation, which induces calcium flux that activates CN. Treatment of human monocytes with either the fungal ligands curdlan, zymosan, and Pam3CSK4 or ionomycin (as a positive control of calcium influx) resulted in increased calcium flux (Fig. 2A). Further quantitative (Fig. 2B) and qualitative (Fig. 2C) analyses confirmed the translocation of NFAT1 to the nucleus after stimulation with Pam3CSK4 and zymosan or zymosan with ionomycin. NFAT translocation was specifically inhibited by CsA in both conditions (Fig. 2B and C).

**Figure 3:**
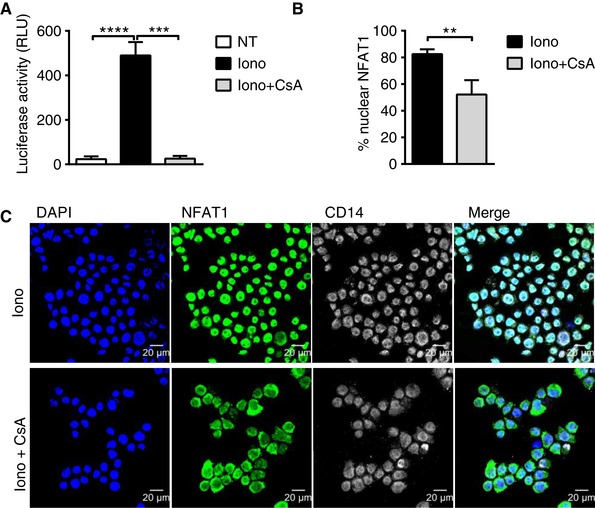
**Functionality of the CN–NFAT pathway in the human monocytic THP‐1 cell line**. **A**) Measurement of luciferase activity following 6 h activation of the NFAT‐luciferase reporter by ionomycin treatment of THP‐1 cells in the presence (Iono+CsA) or absence (Iono) of CsA. Data were analyzed by RM one‐way ANOVA followed by Tukey's multiple comparisons test (^*^ *P* < 0.05, ^**^ *P* < 0.01). Bars indicate the means ± sd. **B**) Proportion of NFAT1 nuclear localization measured as a percentage of nuclear intensity relative to the total cell area fluorescence signal in ionomycin‐treated THP‐1 cells in the presence or absence of CsA. Data were analyzed by Mann‐Whitney test (^*^ *P* < 0.05, ^**^ *P* < 0.01). Bars indicate the means ± sd. **C**) Fluorescence microscopy images showing NFAT1 translocation to the nucleus 30 min after treatment of THP‐1 cells with ionomycin in the presence or absence of CsA

To directly assess the ability of NFAT to induce gene transcription following nuclear translocation in human myeloid cells, we established a NFAT luciferase reporter using the human monocytic THP‐1 leukemia cell line. In this cell line, NFAT activation and translocation leads to initiation of luciferase gene expression through an NFAT binding site in the luciferase promoter. We measured a significant increase in luciferase activity following treatment of THP‐1 reporter cells with ionomycin, which was inhibited by CsA treatment (Fig. 3A). We further confirmed this observation by showing NFAT1 translocation from the cytoplasm to the nucleus after 30 min of ionomycin treatment, which was similarly inhibited by CsA (Fig. 3B and C).

Taken together, these data show that human monocytes express receptors for NFAT‐activating ligands as well as NFAT family members, which are activated by ionomycin or fungal triggers mainly through TLR2 and dectin‐1 and are capable of initiating NFAT‐dependent gene transcription in a reporter cell line.

### 3.2 CN‐NFAT inhibition impairs the monocyte expression of IL‐10 and TNF‐α

**Figure 4:**
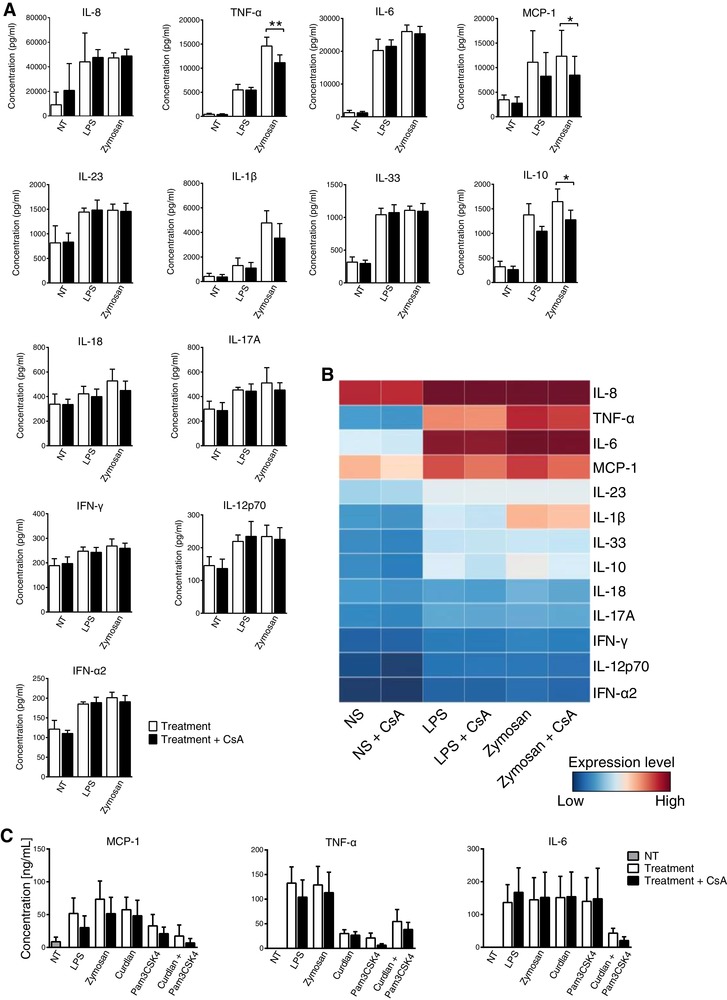
**Influence of CsA treatment on the production of inflammatory cykokines/chemokines by human monocytes in response to PRR ligands**. **A**) Cytokine bead array data show significantly lower secretion of TNF‐α, IL‐10, and MCP‐1 by CsA treated monocytes upon zymosan stimulation. Data are representative of 5 donors. Statistical analysis was performed by RM one‐way ANOVA followed by Tukey's multiple comparisons test (^*^ *P* < 0.05, ^**^ *P* < 0.01). Data represent the means ± sd. **B**) Inflammatory cytokine and chemokine expression levels determined using a 13‐plex cytokine bead array. Each row represents a specific cytokine or chemokine as indicated at the right side of the heat map. Each column represents measurements of these molecules in nontreated control (NT) or stimulated (LPS, zymosan) monocytes in the presence or absence of CsA. **C**) Protein expression of MCP‐1, TNF‐α, and IL‐6 in response to 18 h stimulation with PamCSK4, LPS, or curdlan in the presence or absence of CsA analyzed by ELISA

NFAT‐driven gene expression is activated in murine DCs and macrophages downstream of CD14/TLR4 or dectin‐1 binding by their cognate pathogen‐associated ligands, leading to proinflammatory cytokine expression.^4^ Given the expression of CD14, TLR4, dectin‐1, and NFAT in primary human monocytes shown above, we next asked about the role of NFAT in their cytokine responses to LPS and zymosan. We compared the protein level expression of a panel of 13 inflammatory cytokines and chemokines in freshly isolated human monocytes either treated or not with CsA (Fig. 4A and B). We observed significantly decreased production of zymosan‐induced TNF‐α, IL‐10, and MCP‐1 (*P* < 0.05) under CsA treatment, whereas, as expected, NF‐κB‐regulated cytokine IL‐6 expression remained unchanged. To further understand the involvement of different TLRs and dectin‐1, we analyzed the protein expression level upon triggering with LPS, zymosan, PamCSK4, curdlan, and Pam3CSK4 in combination with curdlan in the presence or absence of CsA (Fig. 4C). Here, we observed a similar trend in the secretion of specific cytokines (MCP‐1 and TNF‐α) after CN‐NFAT inhibition. Together, these data demonstrate that TLR2, TLR4, and dectin‐1 are involved in the NFAT‐dependent expression of several immunoregulatory molecules.

### 3.3 Expression of PTX‐3 in human monocytes is dependent on CN‐NFAT signaling

**Figure 5:**
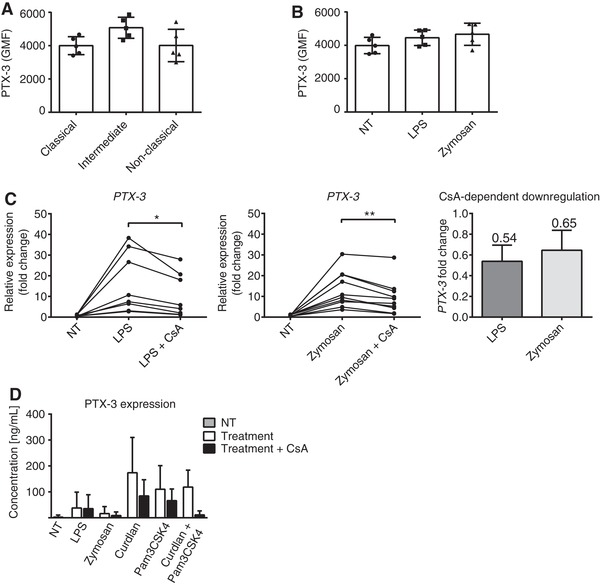
**Expression of PTX‐3 in human monocytes**. **A**‐**B**) Flow cytometric measurement of intracellular PTX‐3 protein level in human monocyte subsets at steady‐state (A) and following 4 h of stimulation with either zymosan or LPS (**B**). (**C**) PTX‐3 mRNA expression measured by qPCR. CsA‐dependent PTX‐3 down‐regulation was calculated in LPS and zymosan treated samples as the ratio between CsA treated and nontreated samples. (**D**) Secretion of PTX‐3 by monocytes stimulated with a variety of PRR ligands, as measured by ELISA. Monocytes were pre‐treated or not with CsA and then stimulated with PRR ligands for 4 h. Data are representative of 7–9 donors. Statistical analysis was performed by RM one‐way ANOVA followed by Tukey's multiple comparisons test (^*^ *P* < 0.05, ^**^ *P* < 0.01). Data represent the means ± sd

We recently identified decreased PTX‐3 expression in murine APCs with impaired CN‐NFAT signaling.^26^ We thus investigated the regulation of PTX‐3 expression in human monocytes and its relationship to NFAT activation. All monocyte subsets expressed intracellular PTX‐3 in the steady state (Fig. 5A). We then stimulated total human blood monocytes with LPS or zymosan and assessed PTX‐3 expression by flow cytometry and qPCR (Fig. 5B and C). Although intracellular protein levels of PTX‐3 were unaffected by LPS or zymosan stimulation (Fig. 5B), we detected marked increases in expression at the mRNA level, which were significantly reduced by CsA treatment (Fig. 5C); the same pattern was evident in the levels of secreted PTX‐3 protein in monocyte supernatants after 4 h of incubation with zymosan, curdlan, and Pam3CSK4 (Fig. 5D). Thus, human monocytes respond to a variety of PRR ligands by increasing transcription and secretion of the antifungal protein PTX‐3 in an NFAT‐dependent manner.

**Figure 6:**
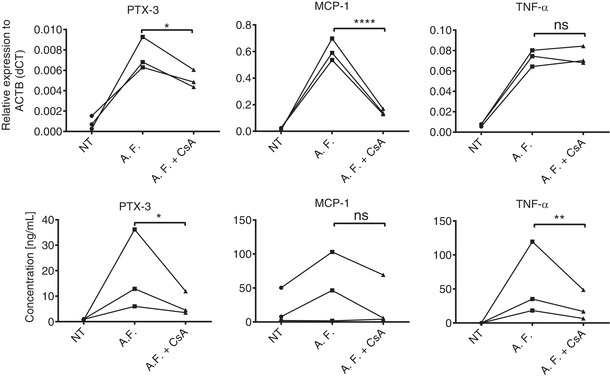
**Heat inactivated *A. fumigatus* conidia initiates PTX‐3 expression through activation of the CN–NFAT pathway**. mRNA (**A**) and protein (**B**) expression was analyzed in human monocytes upon 4 h or 18 h stimulation with heat inactivated *A. fumigatus* (A.F.) conidia in the presence of absence of CsA. Statistical analysis was performed by ratio paired *t* test (^*^ *P* < 0.05, ^**^ *P* < 0.01). Data represent the means ± sd

Because soluble PRR PTX‐3 is important in the defense against fungal pathogens, we validated our findings using heat inactivated *A. fumigatus* conidia. Monocytes responded to *A. fumigatus* infection by increasing the expression of PTX‐3, MCP‐1, and TNF‐α at the mRNA and protein level, which were further reduced by CsA treatment (Fig. 6A and B). Overall, these findings show that an immune response against fungal cell wall components and *A.fumigatus* conidia trigger PTX‐3, MCP‐1, and TNF‐α expression partially in an NFAT‐dependent manner.

### 3.4 In silico analysis of PTX‐3 sequence homology and NFAT1 binding sites in human and murine PTX‐3

**Figure 7:**
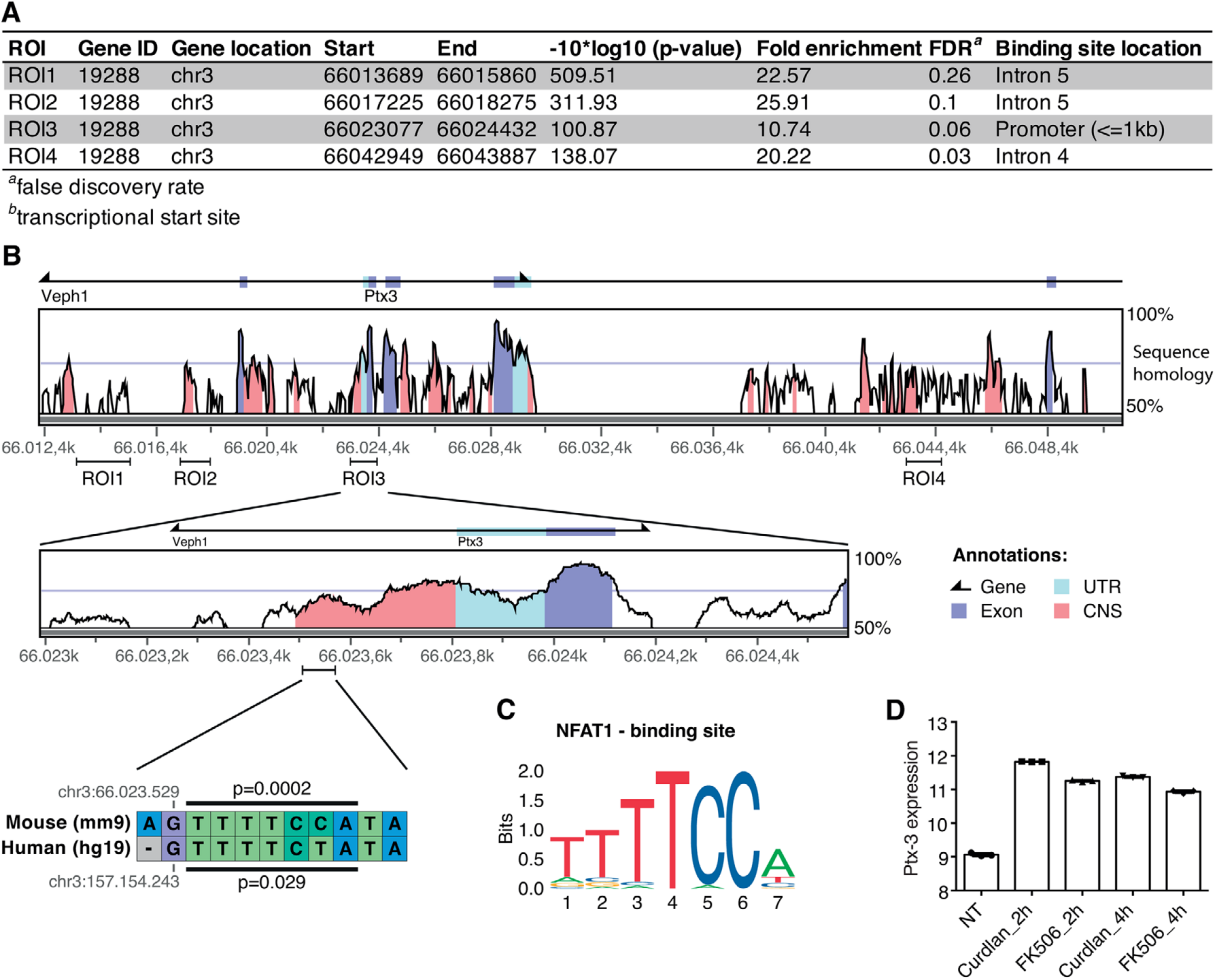
**Analysis of *PTX‐3* sequence homology and NFAT1 binding sites in human and murine *PTX‐3***. **A**.) Re‐analysis of ChIP‐seq data^14^ revealed 4 NFAT1 binding sites in the mouse *PTX‐3* gene (ROI1‐4). **B**) Murine and human sequences for the *PTX‐3* gene were aligned and assessed for sequence homology. ROI3 showed the highest homology and revealed a putative NFAT1 binding site in a conserved noncoding sequence (CNS) ∼300 bp upstream the 5′UTR region. **C**) The NFAT1 binding site sequence obtained from JASPAR 2018 and used for the analysis. **D**) Re‐analysis of gene expression array data identified *PTX‐3* as significantly differentially expressed in murine DCs stimulated with curdlan for 2 h or 4 h compared to either tacrolimus‐treated counterparts (FK506) and nontreated controls. Data represent the means ± sd

The human and mouse *PTX‐3* gene is localized on chromosome 3 and shares high sequence homology.^41^ The promoter region of *PTX‐3* contains multiple transcription factor binding sites including an NF‐κB site, binding sites for activator protein 1 (AP‐1), AP‐2, specificity protein (Sp1), and a gamma IFN activation site^42^; however, it is unknown whether it also contains a binding site for NFAT. To partially unravel the mechanisms underlying CN‐NFAT‐regulated PTX‐3 expression, we reanalyzed available ChIP‐seq data of NFAT1 targets in curdlan‐activated murine DCs,^14^ revealing four NFAT1 binding sites in the murine *Ptx‐3* gene (Fig. 7A, ROI1‐4). To link these data to our experimental results, we aligned the murine and human sequences of the *PTX‐3* gene to evaluate sequence homology. Consistently with previously published data,^41^ the two sequences shared a high degree of conservation, especially in the region surrounding the *PTX‐3* 5′‐UTR (untranslated region; Fig. 7B). Interestingly, this highly conserved region was bound by NFAT1 in curdlan‐activated murine DCs (ROI3),^14^ suggesting the presence of a putative NFAT1 binding site also in the human sequence. To confirm this hypothesis, we screened both sequences for the presence of a NFAT1 binding site and identified a putative common binding motif in a conserved noncoding sequence (CNS) ∼300 bp upstream *PTX‐3* 5′ UTR (Fig. 7B and C). Interestingly, in the same study^14^ *Ptx‐3* was also differentially expressed in curdlan‐stimulated murine DCs compared to untreated control; reanalysis of expression data showed that *Ptx‐3* expression was down‐regulated in DCs stimulated with curdlan in the presence of the CN‐NFAT inhibitor Tacrolimus/FK506 (Fig. 7D). Indeed, these findings of a putative NFAT binding site in human *PTX‐3* promoter are only preliminary and need to be further validated.

In summary, we show that human peripheral blood monocytes up‐regulate *PTX‐3* transcription and secretion following exposure to PRR ligands, under the control of NFAT, as evidenced by inhibition of *PTX‐3* up‐regulation by the CN inhibitor CsA. These findings parallel previous data from a murine model of genetic CN impairment in DCs. Importantly, we also show that *PTX‐3* expression is co‐regulated by CN‐NFAT pathway upon the stimulation of monocytes with *A. fumigatus* conidia, suggesting the importance of the CN‐NFAT signaling axis in human monocytes during infections. We furthermore suggest the likely molecular basis of this correlation by showing that the murine and human *PTX‐3* promoters are potential targets of NFAT1.

## 4 DISCUSSION

In this study we show the presence and functionality of the CN–NFAT signaling pathway in human monocytes obtained from peripheral blood of healthy donors. Stimulation of their PRRs with LPS, zymosan, curdlan, Pam3CSK4, or *A. fumigatus* resulted in CN–NFAT‐dependent changes in their immune gene expression programme, and specific inhibition of the CN–NFAT pathway led to reduced induction of PTX‐3 expression, as previously observed in a mouse model.^26^

Although tissue‐specific genetically engineered mouse models have collectively provided good evidence on the specific functions of CN–NFAT DCs and macrophages, the potential role of CN–NFAT signaling in human monocytes was unknown. Studies in mice have clearly shown that CN expression in CD11c and LysM expressing cells is required for resistance to *A. fumigatus* infection,^26^ likely due to the pathologic Th17 response seen in the lungs of mice lacking the NFAT‐dependent cytokine IL‐2 in CD11c^+^ cells during aspergillosis.^12^ These findings indicate the role of myeloid cells in mice, whereas conclusive results confirming the processes in humans are lacking. CN inhibitors also influence multiple functions in human immune cells including: TNF‐α production^21^ and control of hyphal growth^18^ in macrophages exposed to *A. fumigatus*, and neutrophil antifungal^43^ and antibacterial^44^ activities. Monocytes respond rapidly to the presence of microbial and fungal pathogens by secreting cytokines, chemokines, and antimicrobial factors.^45^ Despite the key role of blood monocytes in orchestrating immunity to fungal pathogens, the role of the CN–NFAT pathway has not been studied in these cells.

The importance of understanding the impact of CN inhibitors on susceptibility to fungal infection is clear: pulmonary aspergillosis is a leading cause of mortality in transplant patients.^7^ Increased risk of severe/invasive aspergillosis in human HSCT recipients has been linked with impaired dectin‐1 signaling^23^ and genetic deficiency in PTX‐3^28^; in this study we consider these findings in the context of the blood monocyte compartment. We show that monocytes express the fungal PRR dectin‐1, and that ligation of this receptor by fungal cell wall components or heat‐inactivated *A. fumigatus* leads to CN–NFAT dependent expression of multiple cytokines, and increased secretion of PTX‐3. Overall, exposure of monocytes to clinical CN‐inhibitor CsA markedly decreased their antifungal responses.

We also noted that human monocytes up‐regulated gene expression of PTX‐3 in response to the bacterial PRR ligand LPS, and that this process was similarly restricted by CN inhibition. Whereas PTX‐3 has been primarily studied for its antifungal role, recent findings have identified it as a diagnostic marker and protective protein during sepsis.^46^, ^47^ Immunosuppressive treatment further increases risks of severe sepsis consequences,^48^, ^49^, ^50^, ^51^ which is particularly intriguing given the pivotal role of monocytes in sepsis,^45^, ^52^ and the results presented here. Further studies on the function of monocyte‐produced PTX‐3 during bacterial infection and the effects of CN inhibition in this context are now warranted.

Independently of the CN‐NFAT role, a therapeutic use for PTX‐3 has been suggested in the context of antifungal therapies.^53^ Furthermore strong research efforts have been made to validate the increased levels of PTX‐3 as a biomarker for severe sepsis^54^, ^55^ and other chronic inflammatory disorders.^56^ These studies have identified PTX‐3 as a very important factor of innate responses.

Studies in mice have proposed several possible molecular mechanisms of CN‐NFAT action in myeloid cells; however, interpreting these results is complicated by the large number of genetic targets of NFATs and their largely unknown effects on myeloid cell functions. There is also evidence of interactions between NFAT members and other transcription factors: Pang et al. recently showed cross‐talk between the NFAT and NF‐κB pathways in coordinately trans‐activating the murine response to *Pseudomonas aeruginosa* infection^57^, whereas several other inflammatory genes including IL‐1α, IL‐1β, IL‐17D, and IL‐22, but also IL‐12β and IL‐23α are known to be expressed under the control of both NFAT and NF‐κB.^14^, ^58^, ^59^ Further studies are necessary to fully understand the crosstalk of both transcription factors in the inflammatory response of myeloid cells, particularly in the human setting.

In summary we provide evidence that NFAT pathways are involved in the expression of the antifungal protein PTX‐3, the inflammatory cytokines IL‐10 and TNF‐α, and the chemokine MCP‐1 during PRRs triggering and *A. fumigatus* infection in human monocytes. Taken together, the data presented here alongside the previous work of Herbst et al. and our own group,^4^, ^9^, ^12^, ^21^, ^26^ make a compelling case for a paradigm‐shift in the way we consider the high susceptibility of transplant patients to fungal infection: the primary responsibility for increased risk of severe fungal infection during treatment with CN inhibitors most likely lays with the inhibition of CN‐driven functions of myeloid cells, and not inhibition of the adaptive immune response.

## DISCLOSURES

The authors declare no conflicts of interest.
